# *Laccaria bicolor* adapts to phosphate deficiency at the developmental, transcriptional and metabolic levels

**DOI:** 10.1007/s00572-025-01236-1

**Published:** 2025-12-11

**Authors:** Anita Loha, Sami Bouziri, Maria V. Aparicio Chacon, Giovanna Ambrosini, Katharina Gutbrod, Peter Dörmann, Minna Kemppainen, Francis Martin, Yves Poirier

**Affiliations:** 1 Department of Plant Molecular Biology, Biophore, University of Lausanne, 1015 Lausanne, Switzerland; 2 Bioinformatic Competence Center, University of Lausanne, 1015 Lausanne, Switzerland; 3 Bioinformatic Competence Center, Ecole Polytechnique Fédérale de Lausanne, 1015 Lausanne, Switzerland; 4 Institute of Molecular Physiology and Biotechnology of Plants, University of Bonn, 53115 Bonn, Germany; 5 Laboratory of Molecular Mycology, Department of Science and Technology, Institute of Basic and Applied Microbiology, National University of Quilmes (UNQ), and National Scientific and Technical Research Council (CONICET), B1876BXD Bernal, Argentina; 6 Université de Lorraine, UMR ‘Interactions Arbres/Microorganismes’, INRAE, INRAE Grand Est – Nancy, 54280 Champenoux, France

**Keywords:** *Laccaria bicolor*, Ectomycorrhizal fungi, Phosphate, Transcriptome

## Abstract

The amount of soluble inorganic phosphate (Pi) in soils is typically low and limiting plant growth. Roots of trees in several forest ecosystems form association with ectomycorrhizal (ECM) fungi, where fungi forage and supply inorganic nutrients, such as Pi, in exchange for fixed carbon. While adaptations of model fungi, such as *Saccharomyces cerevisiae*, to Pi deficiency has been extensively studied, much less is known about how mycorrhizal fungi adapt to Pi deficiency. This study aimed to decipher how the free-living ECM *Laccaria bicolor* mycelium adapts to Pi deficiency. *L. bicolor* grown for 7 days in medium without Pi showed very low Pi and polyphosphate reserves and displayed less compact colonies with spreading hyphae. Pi deficiency resulted in approximately 1500–2000 genes being up- and down-regulated more than 2-fold compared to mycelium grown with abundant Pi, with most genes partially reverting their expression pattern in cultures spiked with Pi for 24 hours. Numerous genes involved in Pi mobilization from organic sources, such as phosphatases and ribonucleases, were induced by Pi deficiency, as well as genes involved in Pi transport, and such expression patterns correlated with increased enzymatic activities. Pi deficiency also induced the synthesis of the betaine lipid diacylglyceryl-*N*,*N*,*N*-trimethylhomoserine (DGTS). Several genes induced by mycorrhization, such as those encoding protease inhibitors belonging to the mycocypin family and Mycorrhizae-Induced Small Secreted Peptides (MiSSP), were also induced by Pi deficiency. Altogether, this study shows that *L. bicolor* can robustly respond to Pi deficiency and identifies parallels between these adaptations and those involved in mycorrhization.

**Supplementary Information:**

The online version contains supplementary material available at 10.1007/s00572-025-01236-1.

## Introduction

Phosphorus (P) is an essential element for life, being found in numerous important molecules such as nucleic acids, lipids, proteins and carbohydrates. P is one of the most limiting nutrients for plant growth in both agricultural and natural ecosystems (Roy et al. 2016; Sattari et al. 2016). Plants only acquire P as soluble inorganic phosphate (H_2_PO_4_^−^). While P is abundant in most soil, it is typically unavailable because it is either present in organic forms, such as phytate, or precipitated in poorly soluble complexes with either calcium or oxides and hydroxides of iron or aluminum. Plants have thus evolved numerous strategies to sense Pi limitation and adjust their metabolism to improve Pi acquisition from the soil and optimize its use for growth and reproduction (Poirier et al. 2022). For example, under Pi deficiency, plants increase secretion of phosphatase by the root system to mobilize Pi from organic sources, increase the expression of phosphate transporters, and modify its metabolism to optimize internal Pi use (Dissayanaka et al. 2021). Another important strategy for enhancing Pi acquisition is by associating with mycorrhizal fungi, including both arbuscular mycorrhizae (AMF) and ectomycorrhizae (ECM). In this symbiosis, the fungi obtain sugars and other essential molecules – such as lipids in the case of AMF - from the plant in exchange for Pi, which is acquired by the extensive hyphal network that explores the soil and mobilizes both organic and inorganic sources of P into Pi (Bonfante and Perotto 1995). In temperate and boreal forest ecosystems, the association of roots of conifers and deciduous trees with ECM is predominant and provides large contributions to the nitrogen and P nutrition of trees, as well as to the carbon cycling (Nehls and Plassard 2018). The development of molecular methods to genetically transform *Laccaria bicolor* along with the sequencing of its genome has made it an excellent model for ECM research (Kemppainen et al. 2005; Martin et al. 2008).

The limited availability of soluble Pi is a challenge not only for plants but also for many organisms living in the soil or in the rhizosphere, including fungi. Adaptation to Pi limitation in fungi has been particularly well studied in the unicellular model *Saccharomyces cerevisiae* (Austin and Mayer 2020; Mouillon and Persson 2006; Secco et al. 2012). This fungus adapts to phosphate deficiency by activating the PHO pathway, primarily regulated by the Pho4 transcription factor. Under low phosphate conditions, the Pho85-Pho80 kinase complex is inhibited, allowing Pho4 to enter the nucleus and activate the expression of numerous genes involved in Pi acquisition, including genes encoding acid phosphatases and phosphate transporters. Inositol pyrophosphates are key molecules regulating this pathway (Austin and Mayer 2020). Beyond *S. cerevisiae*, adaptation to low Pi availability has also been studied in some other ascomycetes, such as *Schizosaccharomyces pombe*, *Candida albicans*, and more prominently *Neurospora crassa* (Bhalla et al. 2022; Tomar and Sinha 2014). However, our understanding of the adaptation to Pi deficiency in mycorrhizal fungi is rudimentary. While numerous studies have examined how the plant-mycorrhizal symbiotic relationship is modulated in response to external Pi levels (Balzergue et al. 2013; Balzergue et al. 2011; Smith et al. 2011), research directly addressing the adaptive responses of mycorrhizal fungi to Pi deficiency remains limited and is frequently centered on phosphate transporter systems (Sportès et al. 2025). In ECM fungi, Pi deficiency is known to trigger the up-regulation of genes encoding Pi transporters belonging to the PHT1 family in *Hebeloma cylindrosporum*, *Boletus edulis* and *Tricholoma sp* (Kothe et al. 2002; Tatry et al. 2009; Wang et al. 2014). A recent transcriptomic study on the ECM fungus *Paxillus involutus* examining hyphae growing under low Pi condition, both in symbiosis with its host tree *Pinus sylvestris*, or asymbiotically, showed that nearly 2000 genes were differentially expressed, including the up-regulation of Pi transporters (Paparokidou et al. 2021). In contrast, transcriptomic analysis of free-living *L. bicolor* mycelium exposed to various Pi concentrations reported that low Pi levels did not significantly affect gene expression, whilst high Pi altered the expression of only 15 genes (Ruytinx et al. 2021). It has been suggested that the lack of transcriptomic response to Pi deprivation in *L. bicolor* may be attributed to a very efficient homeostatic system that quickly redistributes internal Pi stores, or that adaptation to Pi deficiency may be primarily regulated at the post-transcriptional or post-translational level.

In this work, the response of free-living *L. bicolor* mycelium grown in medium with and without Pi was assessed at the level of morphology, P reserves, transcriptome and changes in metabolism. The data shows that Pi deficiency in *L. bicolor* leads to large changes in the transcriptome, which directly impact the mobilization and transport of Pi as well as the membrane lipid composition, and modulate the expression of protease inhibitors belonging to the mycocypin family, transcription factors, and Mycorrhiza-Induced Small Secreted Peptides (MiSSP).

## Material and methods

### Fungal culture maintenance

Dikaryotic strain *L. bicolor* S238N (Maire) P.D. Orton was grown and maintained on solidified P5 agar media containing 2.7 mM diammonium tartrate, 7.35 mM KH_2_PO_4_, 2.0 mM MgSO4, 29.6 μM MnSO_4_, 137 μM H_3_BO_3_, 36.9 μM FeCl_3_, 3.7 μM CuSO_4_, 9.4 μM ZnSO_4_, 0.24 μM (NH_4_)_6_Mo_7_O_24_, 2.97 mM thiamine-HCl, 14.6 mM maltose, 111 mM (D+) glucose, 2.5 mM 2-(N-morpholino) ethanesulfonic acid (MES) and agar (20 g/L), final pH 5.5. Cultures were maintained at 24 °C in dark chamber and re-cultured biweekly. For liquid cultures, *L. bicolor* mycelium was grown in liquid P5 media at 24 °C in dark chamber under 100 rpm shaking conditions. Phosphate-deficient P5 media was made by replacing KH_2_PO_4_ by K_2_SO_4_ to a final concentration of 3.67 mM. Fungal liquid cultures were established by the fragmentation of mycelium grown in solid P5 medium using a sterile stainless-steel disperser blade for 5 sec.

### P content measurement

Fungal mycelium was harvested and washed with P5 media without Pi and dried in an oven at 65 °C for 48 h. Dry biomass was acid-digested with concentrated HNO_3_ at 100 °C overnight. The tubes were next heated at 145°C to completely evaporate the HNO_3_ under a fume hood. After cooling at room temperature, the digested biomass was then heated with 1 N H_2_SO_4_ at 100 °C for 1 hour. Soluble Pi in solution was measured using the molybdate assay (Ames 1966).

### Polyphosphate purification and staining

Polyphosphate (polyP) content in *L. bicolor* mycelium was visualized using the JC-D7 dye (http://www.glixxlabs.com) (Angelova et al. 2014). A solution of 100 μM JC-D7 was applied to fungal mycelium for 1.5 h at 25 °C as described (Zhu et al. 2020). Mycelium was then fixed under vacuum with 4% paraformaldehyde containing 0.1% Triton X-100. The polyP-JC-D7 complex was imaged using the Zeiss LSM 710 confocal microscope with an excitation of 405 nm and an emission window of 480–510 nm. Images were subjected to spectral deconvolution.

Total polyP content was determined as previously described with slight modifications (Trilisenko et al. 2019). Briefly, *L. bicolor* mycelium was collected and washed twice in ice cold 0.9% NaCl solution. The biomass was then frozen, powdered in liquid nitrogen, and polyP was extracted by applying two consecutive treatments of 15 minutes each with 0.5 N HClO_4_ solution at 4 °C under constant shaking. Samples were centrifuged at 3000*g* and the supernatant containing the acid-soluble polyP was conserved. The acid-insoluble polyP remaining in the precipitated biomass was extracted by two consecutive treatments with 0.5 N HClO_4_ at 90 °C for 20 min. All supernatants were combined and treated with 1 N HCl at 100 °C for 10 min, followed by determination of Pi according to the molybdate assay (Ames 1966).

### ^31^P-NMR analysis

Briefly, mycelium from liquid cultures were introduced into 5 mm glass NMR tubes with 10% of D_2_O added for locking and analyzed by 1D ^31^P-NMR spectroscopy at 600 MHz (^1^H frequency) on a Bruker Avance III HD instrument equipped with a cryogenically cooled BBO probe. Spectra were acquired with a basic excitation-measure sequence with ^1^H decoupling. The hard pulse on ^31^P was 18 μs, spectra were centered at 0 ppm and acquired over a spectral width of 70 ppm with 8 k points which makes for an acquisition time of 0.48 s, with an additional interscan delay of 0.8 s. Spectra were processed with 20 Hz of line broadening. Acquisition and processing were done in Topspin v. 3.6.

### Phosphatase assay

The activity of secreted acid phosphatase found in the growth media was measured using para-nitrophenyl-phosphate (pNPP) as a substrate. For acid phosphatase activity, the assay buffer contained 100 mM Tris-HCl pH 5.5, 5 mM MgCl_2_ and 4 mM pNPP, while for alkaline phosphatase activity it contained 100 mM CAPS-Tris pH 9.0, 5 mM MgCl_2_ and 4 mM pNPP. The reaction was stopped by adding 40 μl of 5 N NaOH in a reaction volume of 1 ml and the absorbance was measured at a wavelength of 405 nm. One unit was defined as the activity converting 1 nmol of pNPP to 4-nitrophenol per min at 25 °C.

### Ribonuclease assay

Ribonuclease assay was performed essentially as previously described (Thorn et al. 2012). Briefly, the activity of fungal ribonucleases secreted in the liquid medium was assayed in a buffer containing 0.2 M Na Acetate, 50 mM NaCl, 4 mM EDTA, 4 mg/ml of yeast RNA (#10109223001, Sigma-Aldrich) at a final pH of 4.6. The reaction was stopped by adding 1/4 v/v of a solution made of 0.75% uranyl acetate diluted in 25% HClO_4_. The mixture was cooled on ice, centrifuged at 14000 g at 4 °C for 15 minutes and the supernatant was collected. The amount of short-chain RNA oligomers present in the supernatant was measured with a spectrophotometer at OD_260_. One unit was defined as the amount of small-chain RNA giving an OD260 = 1 per hour at 25 °C.

### Lipid analysis

Total cellular lipids were first extracted from lyophilized mycelia using chloroform:methanol (1:2, v/v) for 4 h at 4 °C followed by a second extraction with chloroform:methanol (2:1, v/v) for 16 h at 4 °C. The two extracts were combined and dried under streaming nitrogen. The dried lipids were dissolved in 200 μl of the Q-TOF solvent chloroform/methanol/300 mM ammonium acetate (300:665:35, v/v/v). 10 μl of lipid extracts were mixed with 10 μl of the internal standard di-16:0-DGTS-d9 (0.07 nmol/μl in chloroform/methanol (2:1, v/v, Avanti Polar Lipids) and diluted with 80 μl of Q-TOF solvent. DGTS was separated using a Nucleodur Gravity column (Macherey and Nagel, 50 × 4.6, 1.8 μm particle size) with an Agilent 1200 Series HPLC with binary pump at a flow rate of 0.8 ml/min. The LC solvents were adapted from Zhang et al. (Zhang et al. 2016). DGTS content was analyzed using an Agilent 6530 Q-TOF LC/MS device in the positive ion mode. Auto-MS/MS and subsequent scanning using the Agilent MassHunter Qualitative Analysis for a fragment characteristic for the headgroup (m/z 236.1509) was used for the identification of all molecular species of DGTS present in *L. bicolor* free-living mycelium. The molecular species identified in this way were analyzed and the peak areas were used for the quantification of DGTS in relation to the peak area of the internal standard.

### Pi import

Pi import into *L. bicolor* mycelium was performed using ^33^Pi-orthophosphate added to the growth medium. Following a 15 min incubation, the fungal mycelium was harvested and washed thoroughly with medium lacking labeled ^33^Pi at 4 °C. The mycelium was weighted and placed into a solution of 5% SDS and heated at 60 °C overnight. Radioactivity was measured using the Revvity Ultima Gold scintillation cocktail and a Tri-Carb 4910 liquid scintillation counter.

### RNAseq

RNA-seq libraries were prepared from 1 μg of total fungal RNA with the Illumina TruSeq Stranded mRNA reagents (Illumina) using a unique dual indexing strategy following the official protocol (https://support.illumina.com/downloads/truseq-stranded-mrna-reference-guide-1000000040498.html). The process was automated on a Sciclone liquid handling robot (PerkinElmer). Libraries were quantified by a fluorometric method (QubIT, Life Technologies) and their quality assessed on a Fragment Analyzer (Agilent Technologies). Sequencing was performed using the Illumina NovaSeq 6000 for 300 cycles (paired-end 150 nucleotides reads) and an average of 40 million reads was obtained per sample. Sequencing data were demultiplexed using the bcl2fastq2 Conversion Software (version 2.20, Illumina).

RNA expression was quantified using Kallisto (v0.48.0) a k-mer counting software that uses pseudoalignments for reducing quantification error and improving speed (Bray et al. 2016). An average pseudo-alignment rate of 90% was achieved. Differential expression analysis of transcripts was performed with Sleuth (0.30.0) (Pimentel et al. 2017). After fitting the model design (design <− ~ Pi_condition), the Wald test was applied to identify significant differences between different phosphate conditions. For normalization of estimated counts size factors were used. The false discovery rate (FDR) <0.05 was set as the threshold for significantly differentially expressed genes (DEGs). All gene searches were matched to the most recent *L. bicolor* genome annotation from the JGI database server (https://mycocosm.jgi.doe.gov/pages/search-for-genes.jsf?organism=Lacbi2) (Martin et al. 2008).

## Results

### Low Pi supply impacts *L. bicolor* growth and intracellular P reserves

**Fig. 1:**
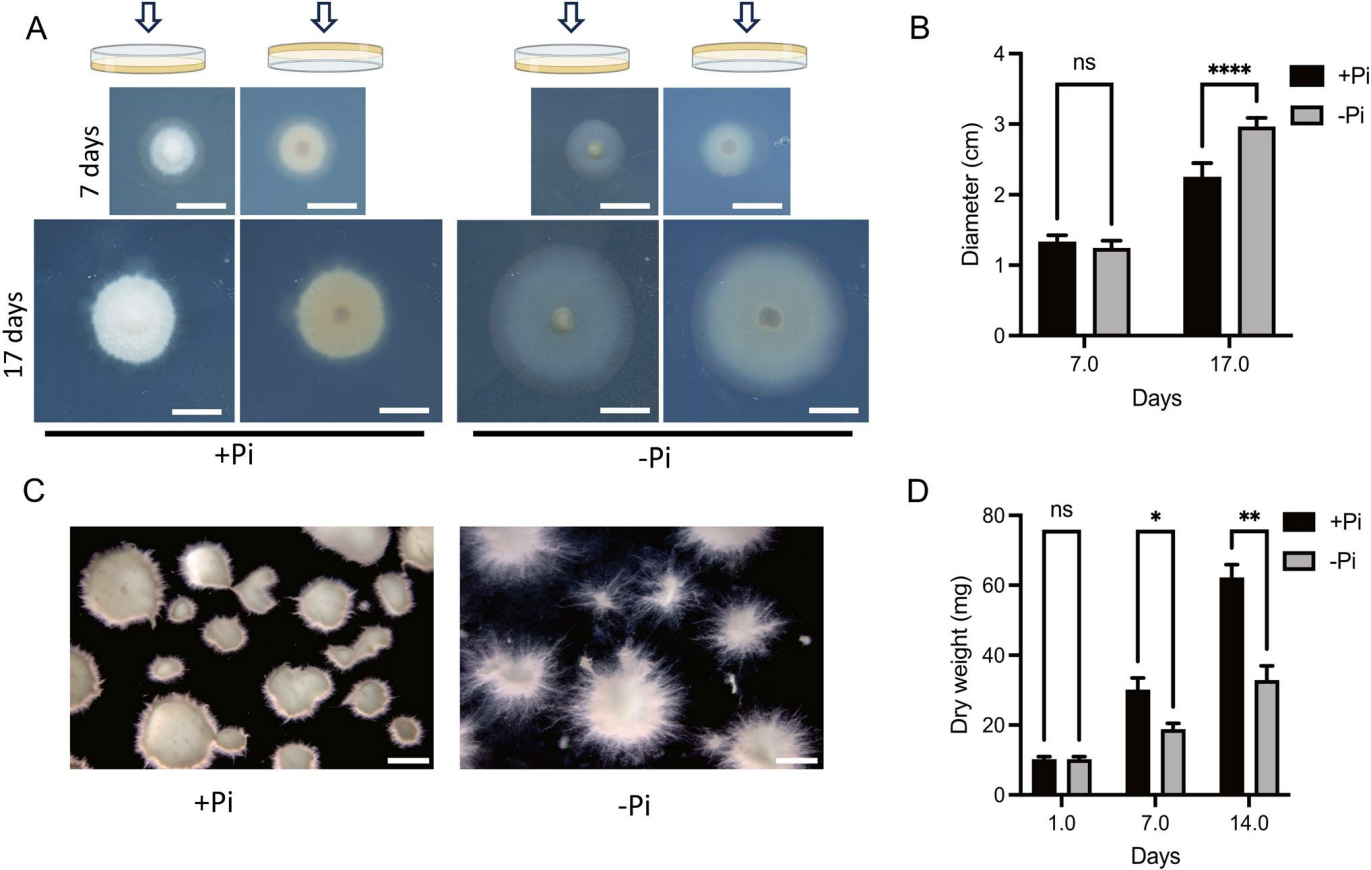
Pi deficiency influences the growth of free-living *L. bicolor* mycelium. (**A**) Pictures of 7- and 17-day-old colonies grown in Pi-sufficient (+Pi) and Pi-deficient (-Pi) solid P5 media. The appearance of the colonies is shown when observed from above or below the petri dish, respectively. Bars, 1 cm. (**B**) Diameter of colonies shown in A. (**C**) Phenotypical traits of 7-day-old colonies growing in +Pi and -Pi liquid P5 media. Bars, 1 cm. (**D**) Dry weight of *L. bicolor* mycelium grown in +Pi and -Pi liquid P5 media after 1, 7 or 14 days. Data are presented as mean ± standard error of the mean (SEM) from three to five independent biological replicates. Statistical analysis was performed using two-way ANOVA, followed by Sidak’s multiple comparisons test. Asterisks indicate statistical significance (*P < 0.05, **P < 0.01, ***P < 0.001, ****P < 0.001)

**Fig. 2:**
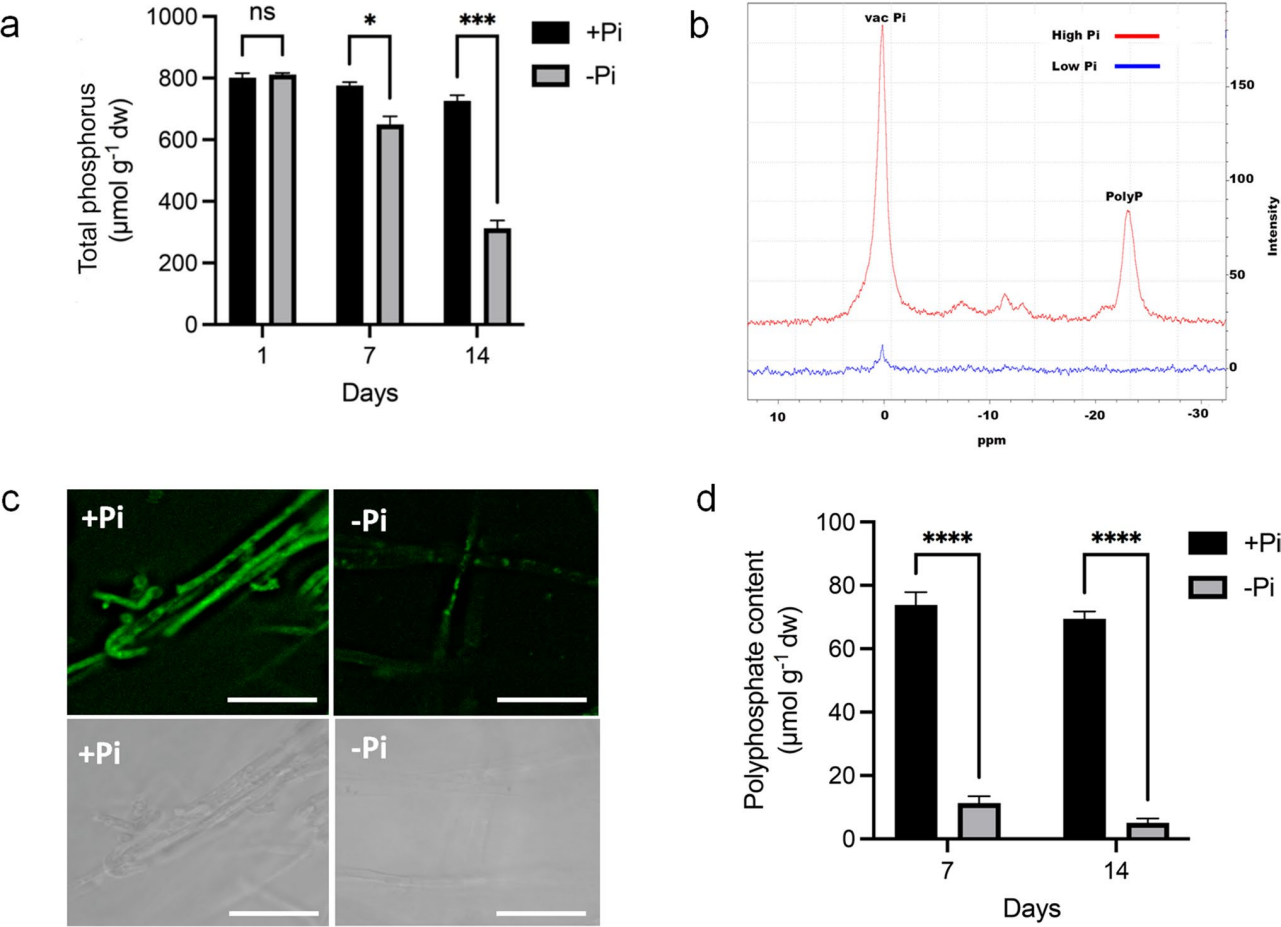
Pi deficiency leads to a decrease in the main phosphorus reserves. (**A**) Total amount of phosphorus in *L. bicolor* mycelium grown in +Pi and -Pi liquid P5 media after 1, 7 and 14 days. (**B**) In vivo ^31^P-NMR of mycelium grown for 7 days in +Pi or -Pi liquid medium. Main peaks representing vacuolar Pi and polyP are shown. (**C**) Detailed confocal imagining of *L. bicolor* hyphae stained with the polyP dye JC-D7 after 7 days of growth in +Pi and -Pi liquid P5 media. Bars, 20 μM. (**D**) Quantification of polyphosphate in mycelium grown in +Pi and -Pi liquid P5 media for 7 days. Data are presented as mean ± standard error of the mean (SEM) from three independent biological replicates. Statistical analysis was performed using two-way ANOVA, followed by Sidak’s multiple comparisons test. Asterisks indicate statistical significance (*P < 0.05, **P < 0.01, ***P < 0.001, ****P < 0.001)

*L. bicolor* mycelium was grown in P5 medium containing 7.35 mM Pi as KH_2_PO_4_ and diammonium tartrate as the sole nitrogen source. For Pi deficiency, KH_2_PO_4_ was replaced by K_2_SO_4_. When grown in Pi-sufficient agar-solidified medium, *L. bicolor* colonies were more compacted and denser compared to colonies grown in Pi-deficient medium. This was more evident after 17 days, where the Pi-deprived colonies exhibited greater diameter due to extended outward growth of hyphae (Fig. 1A-B). Similar phenotypical differences were also observed in mycelium grown in liquid cultures (Fig. 1C). Such cultures grew more slowly in low Pi media than high Pi, resulting in mycelium biomass being significantly different at 7 and 14 days after Pi withdrawal (Fig. 1D). The total amount of P in mycelium grown in -Pi media decreased from approximately 800 μmol/g dry weight (DW) to 300 μmol/g DW after 14 days, while mycelium grown on +Pi showed only a small reduction to 750 μmol/g DW (Fig. 2A). Ionomic analysis by ICP-MS showed that the level of potassium, magnesium, zinc, manganese, copper and iron were not significantly affected by the levels of Pi present in the medium (Supplemental Figure 1). Analysis of mycelium by in vivo ^31^P-NMR showed that growth for 7 days in -Pi medium resulted in a strong reduction in the concentration of both vacuolar Pi and polyphosphate (polyP), the two major P reserves in hyphae (Fig. 2B). Reduction of polyP in hyphae grown in -Pi media could also be visualized with the JC-D7 dye (Fig. 2C). Quantification of polyP in mycelium grown for 7 and 14 days in -Pi medium showed a 7- and 14-fold reduction, respectively (Fig. 2D). Altogether, these data indicate that depletion of P reserves in free-living *L. bicolor* grown in P-deprived medium was accompanied by morphological changes, with reduced mycelium density and greater extension of hyphal growth to maximize the surface exploration of the medium.

### The *L. bicolor* transcriptome strongly responds to pi deficiency

To prepare material for transcriptome analysis, mycelium of *L. bicolor* was grown in liquid P5 supplemented with 7.35 mM Pi or without Pi, for 7 days (HP and LP, respectively). In parallel, one set of fungal cultures was also grown in P5 medium lacking Pi for 7 days and spiked with Pi to a final concentration of 7.35 mM for an additional 24 hours before collection (LPspike). Total RNA was extracted from each replicate for all three conditions and used for preparing RNAseq libraries.

**Fig. 3:**
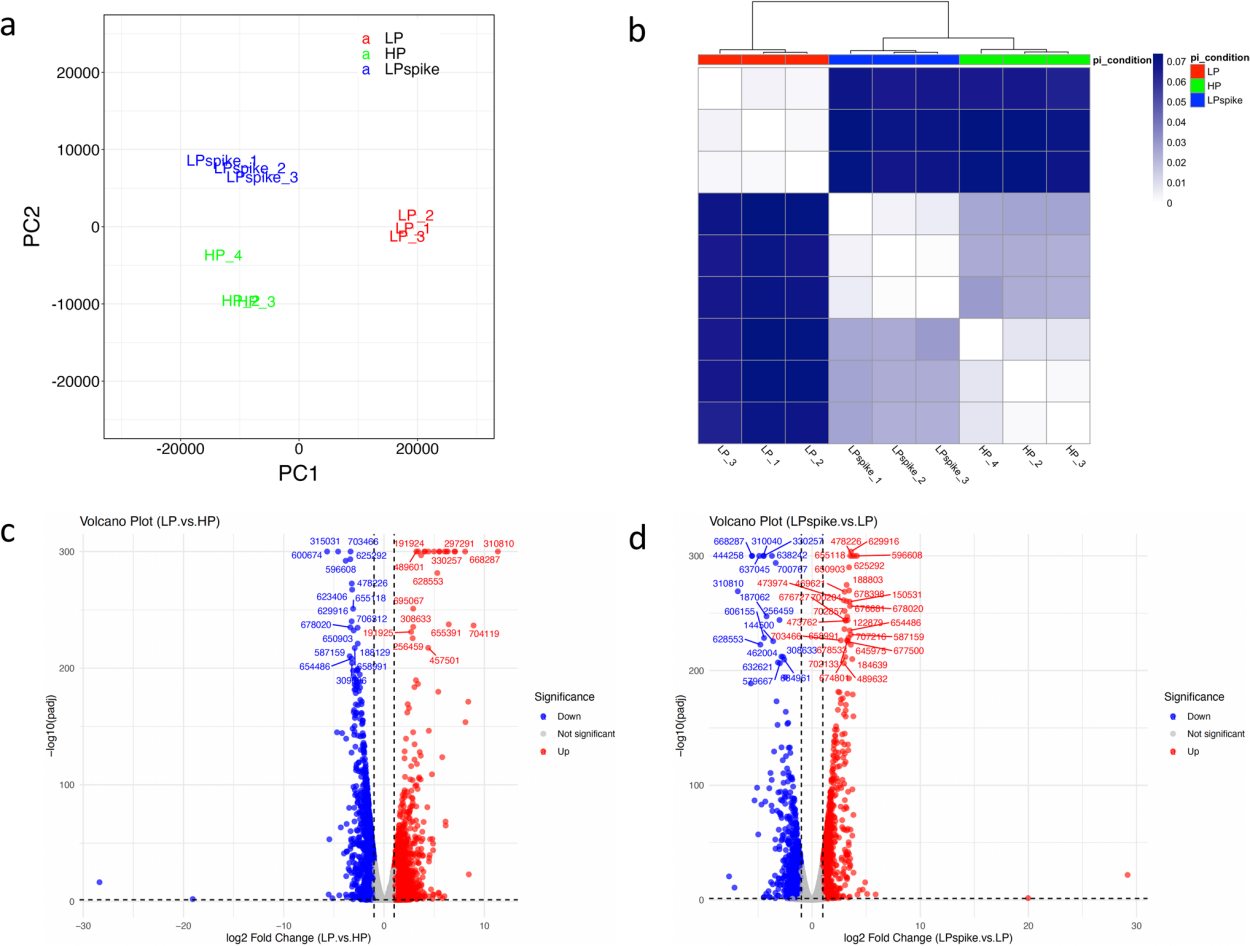
Pi deficiency induces large changes in *L. bicolor* transcriptome. (**A**) Principal component analysis of the RNA-seq samples used in the transcriptome analysis. The units of x and y axes are arbitrary but reflect variances in the data sets. The plot was generated using the Sleuth program (https://pachterlab.github.io/sleuth/). (**B**) Jensen-Shannon divergence analysis of the biological triplicates used in the RNAseq analysis for high Pi (HP), low Pi (LP) and LP samples spiked with Pi for 24 hours (LPspike). (**C**, **D**) Volcano plots displaying the DEGs resulting from the comparison between LP versus HP (**C**) and between LPspike versus LP (**D**)

Principal component analysis of gene expression showed clear separation between the experimental treatments while the biological replicates were grouped together (Fig. 3A). Jensen-Shannon divergence analysis further showed low divergence between the replicates within each specific treatment and a closer expression pattern between the HP and LPspike conditions as compared to the LP, indicating that the addition of Pi for 24 hours to LP cultures enabled a shift in gene expression more similar to the HP control condition (Fig. 3B).

**Fig. 4:**
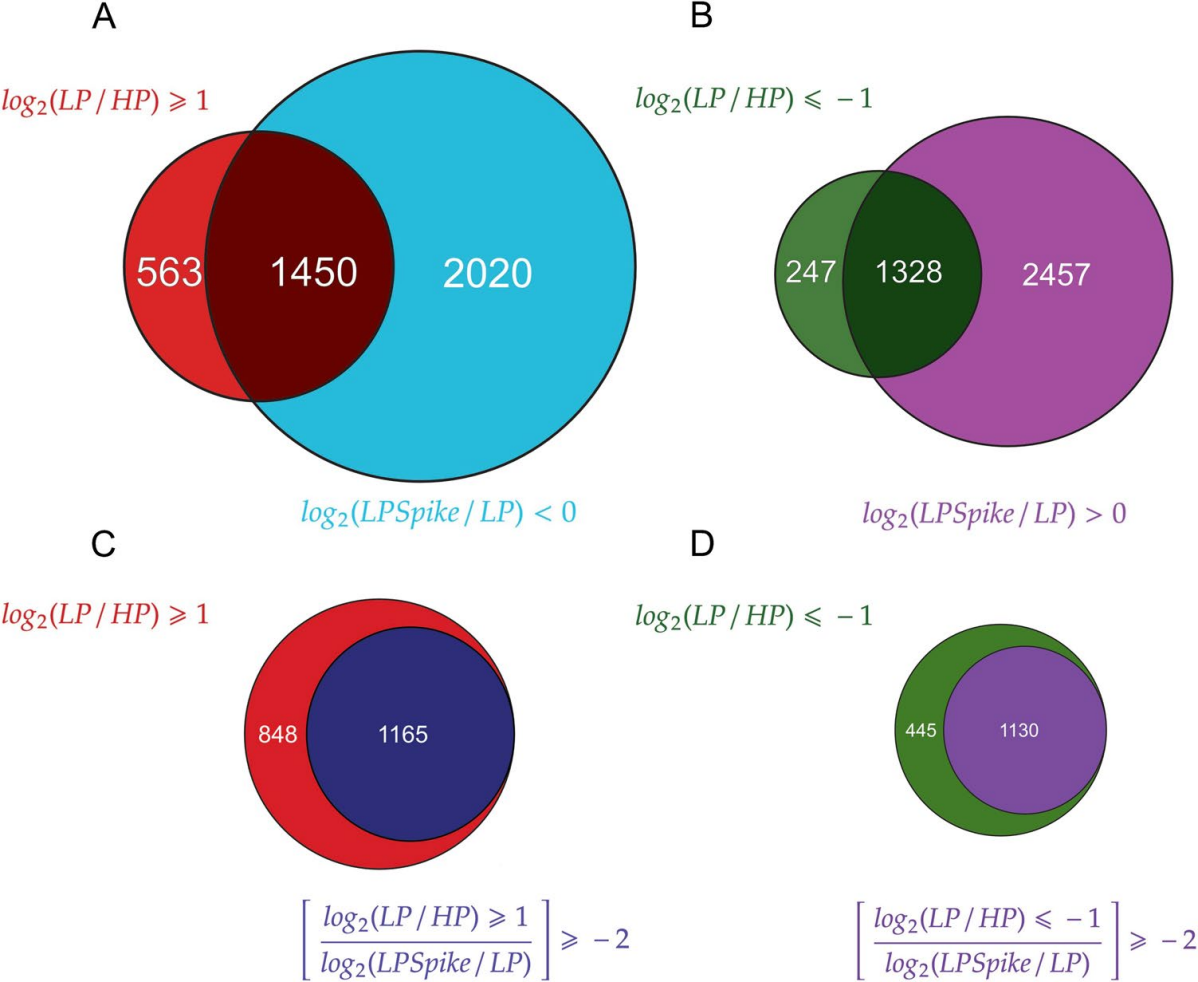
Venn diagrams showing the overlaps between the DEG clusters. (**A**) Genes overexpressed in the LP vs HP comparison with a log_2_ ≥ 1 were compared to genes down-regulated in the LPspike vs LP comparison with a log_2_ < 0. (**B**) Genes under-expressed in the LP vs HP comparison with a log_2_ ≤ −1 are compared to genes up-regulated in the LPspike vs LP comparison with a log_2_ > 0. (**C**) Genes over-expressed (log_2_ ≥ 1) or (**D**) under-expressed (log_2_ ≤ −1) in the LP vs HP comparison which exhibited an inverted expression trend of at least 50% of their log_2_ value in the LPspike vs LP comparaison

There were 10,171 differentially expressed genes (DEG) for the LP vs HP comparison using an adjusted p value threshold of 0.05, while 7255 DEGs were found to be differentially regulated between the LPspike vs LP condition (Supplemental Table 1). The distribution of genes up-regulated (log_2_ ≥ 1) or down-regulated (log_2_ ≤ −1) was visualized in volcano plots for both comparisons (Fig. 3C-D). From the group of 2013 DEGs with a log_2_ ≥ 1 in LP vs HP, a total of 1450 genes (72%) were significantly down-regulated following the Pi spike (Fig. 4A). Similarly, 1328 genes (84%) from the 1575 DEGs with a log_2_ ≤ −1 were significantly up-regulated in response to the Pi spike (Fig. 4B). To assess the impact of the short-term Pi re-supply on the transcriptome, we looked for genes whose expression trends reversed by at least 50% of their initial log_2_-fold change observed under long-term Pi deficient samples. From the initially up-regulated genes in LP, a total of 1165 genes (58%) decreased their expression following the Pi spike, while 1130 (72%) from the initially down-regulated genes by LP showed an increased in expression after Pi addition (Fig. 4C, D). It is concluded from this analysis that a majority of genes differentially expressed by the 7-day LP treatment at least partially reverted their expression following a 24 h treatment with Pi.

**Fig. 5:**
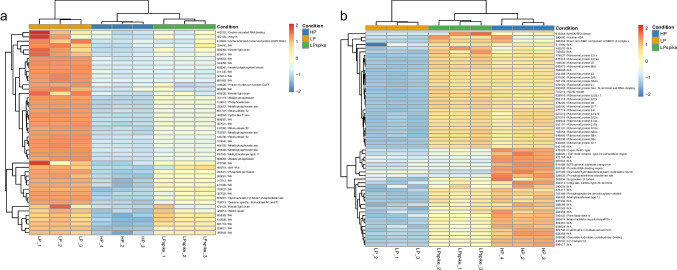
Heatmap clustering of DEGs during Pi deprivation. (**A**-**B**) Heat maps were built using genes up-regulated by a log_2_ ≥ 4 (**A**) and down-regulated by a log_2_ ≤ −3 in the LP versus HP comparison

Analysis was further focused on the genes showing the strongest up- and down-regulation in expression in the LP vs HP comparison. Figure 5 shows the heatmap clustering the 47 and 59 DEGs displaying a log_2_ ≥ 4 and log_2_ ≤ −3, while Table 1 and 2 list the DEGs with a log_2_ ≥ 3 and log_2_ ≤ −3, respectively, and which contain an Interpro domain allowing prediction of protein function. Table 1 shows that Pi deprivation leads to a strong induction of the expression of numerous genes expected to be directly involved in the adaptation to Pi deficiency (shaded in gray). These include several genes encoding secreted proteins involved in scavenging Pi from organic sources, such as phosphatases, phosphodiesterases, ribonucleases and endonucleases. Pi deficiency also induced genes involved in P transport, such as homologs of the *S. cerevisiae* PHO84 Pi transporter (297291 and 191924) and Git1 (677598), involved in the transport of glycerophosphoinositol and glycerophosphocholine, used as sources of inositol and Pi. A gene homologous to the *S. cerevisiae* KCS1 (638242) involved in the synthesis of inositol polyphosphates, implicated in Pi-deficiency signaling, as well as those involved in lipid modification (further discussed below; 637045, 456129 and 667823) were also strongly induced. All these genes, except one (667823), are robustly down-regulated following replenishment of Pi in the LPspike samples.

**Table 1:**
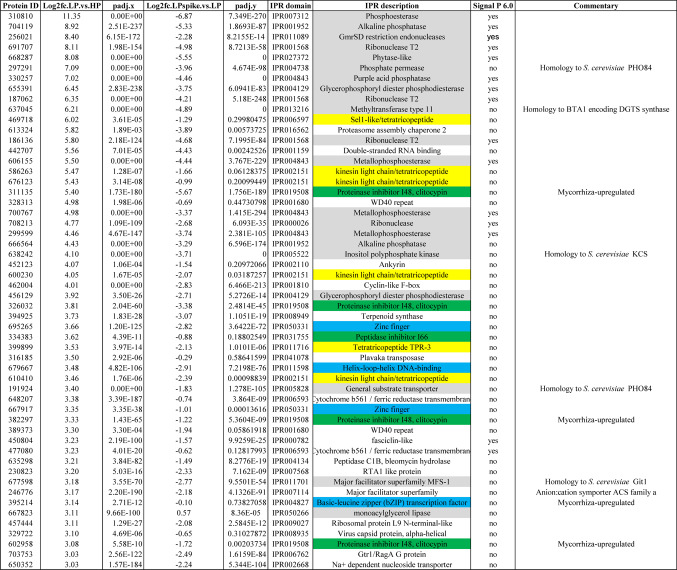
*L. bicolor* genes up-regulated by Pi deficiency with log_2_ ≥ 3

Among the genes strongly up-regulated upon Pi deficiency are 6 encoding proteins containing tetratricopeptide motifs, 5 encoding proteinase inhibitors, including 4 mycocypins, and 4 genes encoding potential transcription factors (in yellow, green and blue, respectively; Table 1). Further analysis showed that expression of 12 out of 19 *L. bicolor* mycocypin gene family members were significantly up-regulated by Pi deficiency, including 6 that were previously reported to be up-regulated upon mycorrhizal associations with *Populus tremula x alba* roots (Plett et al. 2022) (Supplemental Table 2).

Analysis of genes strongly down-regulated by Pi deficiency (Table 2) showed a preponderance (23 out of 44) for genes encoding ribosomal proteins (Table 2, marked in gray), indicating an overall decrease in protein translation. Of note was also the strong down-regulation of a distinct gene with high homology to the *S. cerevisiae* PHO84 (600674). All these genes were also systematically up-regulated upon re-addition of Pi to the medium.

**Table 2.**
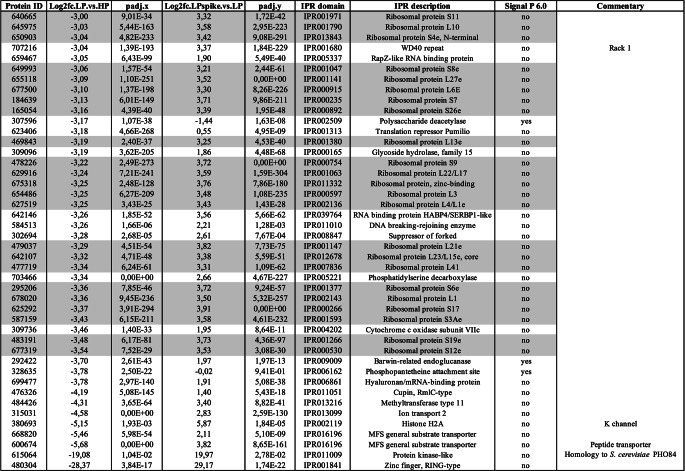
*L. bicolor* genes down-regulated by Pi deficiency with log_2_ ≤ −3

**Fig. 6:**
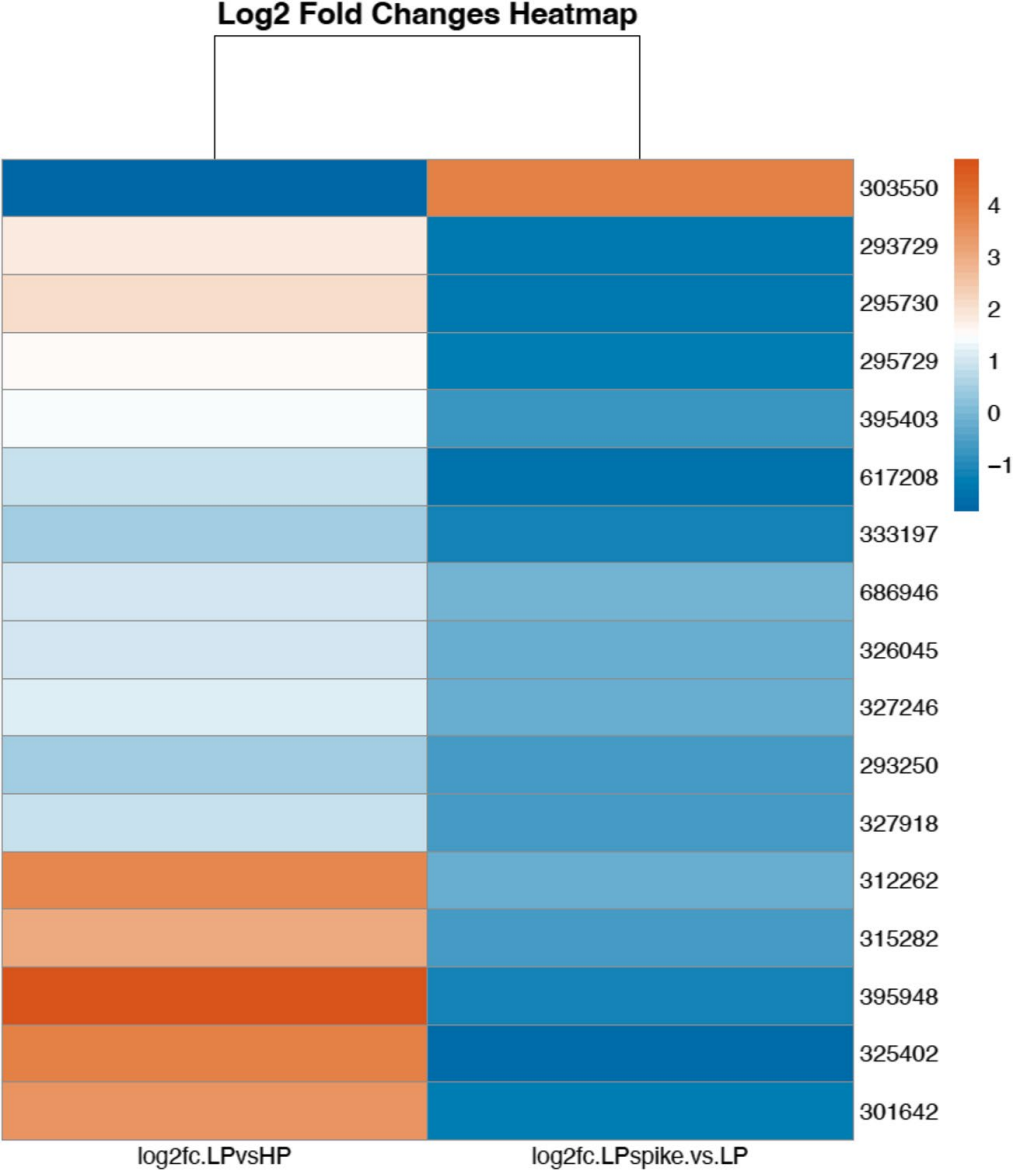
Heatmap of the DEGs encoding for putative MiSSPs identified in the LP vs HP and LPspike vs LP comparisons

Among the list of genes up- (log_2_ ≥ 3) and down- (log_2_ ≤ −3) regulated by Pi deficiency are numerous genes with no Interpro domains that could be used to assign a particular function (Supplemental Tables 3–4; 75 genes up-regulated and 14 genes down-regulated). Such genes were further analyzed for the presence of potential N-terminal signal peptide using both the SignalP 6.0 program (https://services.healthtech.dtu.dk/services/SignalP-6.0/) and InterProScan (https://www.ebi.ac.uk/interpro), as well as the presence of transmembrane helices. From this set, we identified 13 and 1 genes up- and down-regulated, respectively, that were predicted to encode secreted proteins, with 10 proteins having predicted molecular weight lower than 30 kD (Table 3). Five of those genes up-regulated by Pi deficiency were previously annotated as encoding Mycorrhiza-Induced Small Secreted Proteins (MiSSP) (Martin et al. 2008). Figure 6 shows the heatmap for all 17 MiSSP genes with detectable expression in our transcriptomic study, highlighting 5 MiSSP up-regulated (395948, 325402, 312262, 301641 and 315282) and one down-regulated by Pi deficiency (303550).

### *L. bicolor* physiologically adapts to Pi deficiency

**Fig. 7:**
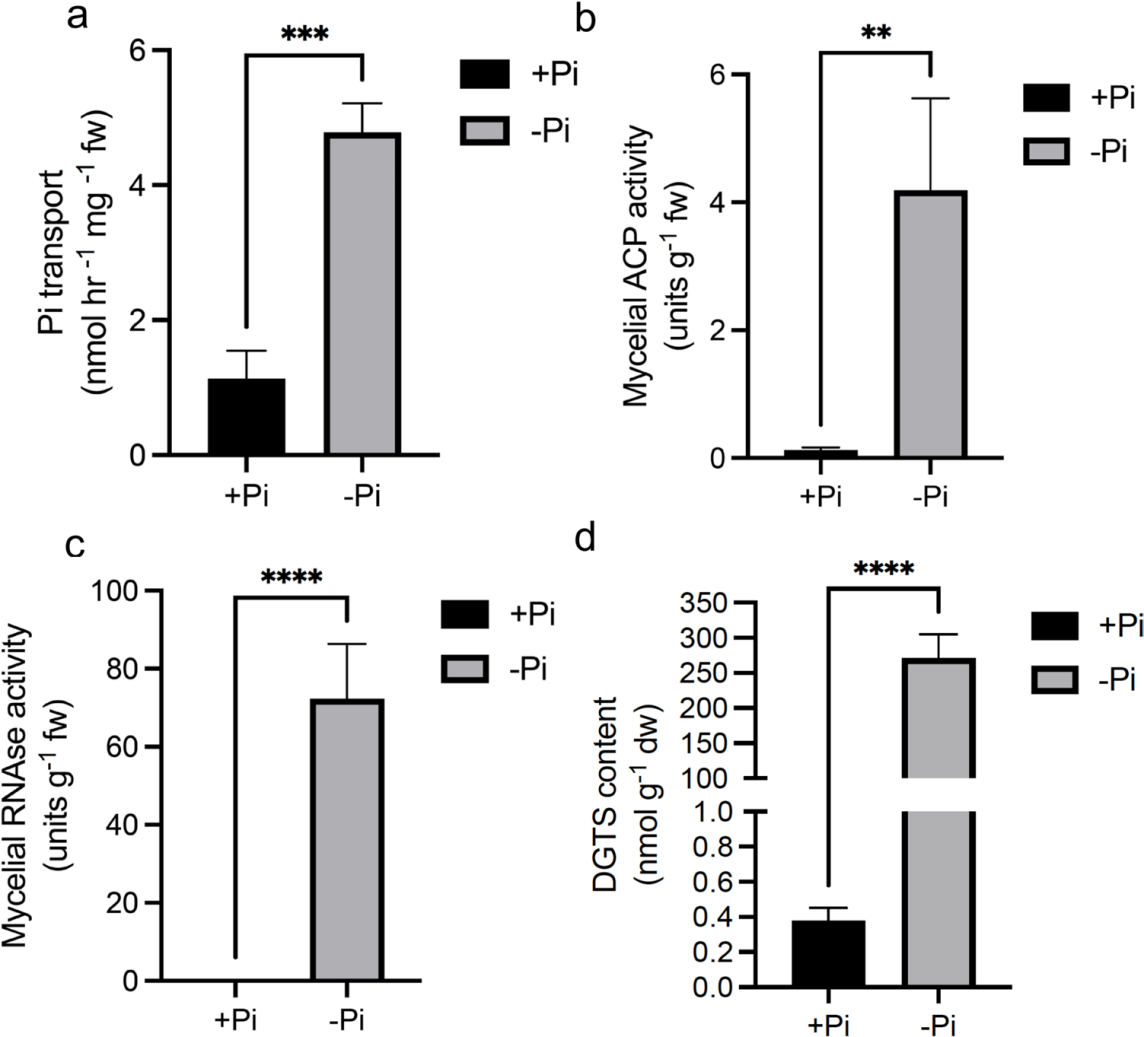
Pi deficiency induces changes in enzyme activities and lipid content in *L. bicolor* mycelium. (**A**) Uptake rate of radiolabeled ^33^Pi in *L. bicolor* mycelium previously grown for 7 days in +Pi and -Pi liquid P5 media. (**B**-**C**) Enzymatic activity of secreted acid phosphatases (**B**) and secreted ribonucleases (**C**). (**D**) Quantification of DGTS content in lipids isolated from *L. bicolor* mycelium after 7 days of growth in +Pi and -Pi liquid P5 media. Data are presented as mean ± standard error of the mean (SEM) from three to five independent biological replicates. Statistical analysis was performed using unpaired t-test. Asterisks indicate statistical significance (*P < 0.05, **P < 0.01, ***P < 0.001, ****P < 0.001)”

We examined whether changes in gene expression triggered by low Pi in the medium impacted the activity of some key enzymes involved in Pi-deficiency adaptation response. The capacity of mycelium to import ^33^Pi from the medium was substantially increased upon Pi deficiency (Fig. 7A). Mycelium grown in low Pi medium also showed a large increase in both ribonuclease activity and acid (pH 5.5) phosphatase activity secreted into the growth medium (Fig. 7B, C). Surprisingly, and despite the strong increased expression of genes annotated as alkaline phosphatase (704119 and 666654), the level of phosphatase activity at pH 9.0 was below detection in our assay.

Transcriptomic analysis showed the strong increased expression of genes involved in lipid synthesis and turnover (Table 1). Gene 637045 encodes a protein with homology to the *Chlamydomonas reinhardtii* BTA1 (Supplementary Figure 2) involved in the synthesis of the betaine lipid diacylglyceryl-*N*,*N*,*N*-trimethylhomoserine (DGTS), a non-phosphorus polar lipid that can functionally replace phospholipids. In accordance with this increase in gene expression under low Pi availability, the content of DGTS-derived lipids in *L. bicolor* membrane showed a large increase upon Pi-deficiency (Fig. 7D).

## Discussion

*L. bicolor* grown under Pi-deficient conditions showed reduced colony density but increased hyphal spreading. Similar phenotypical response to Pi deficiency has been observed in the ECM fungus *Paxillus involutus* (Paparokidou et al. 2021). These data indicate that Pi deficiency induces similar growth adaptations in distinct ECM to favors hyphae elongation, a trait that enhances the fungal ability to explore greater soil volume for P scavenging.

The current work shows that Pi deficiency in free-living *L. bicolor* mycelium triggers profound changes in the transcriptome that are rapidly partially reversed upon Pi re-supply. Among the most up-regulated genes are several encoding phosphatases and nucleases, involved in mobilizing Pi from organic sources. ECM fungi are known to produce various types of secreted phosphatases, and their production can be modulated by the availability of Pi in the soil (Colpaert et al. 1997; Louche et al. 2010; McElhinney and Mitchell 1993). A recent study has shown that cultures of free-living *L. bicolor* secreted approximately the same amount of acid phosphatase when grown in media with or without Pi, although the impact of the low Pi treatment on the mycelium P stores was not assessed (Yuan et al. 2024). Interestingly, no alkaline phosphatase activity could be detected. A few genes encoding phosphatases in *Lactarius deliciosus* and *L. bicolor* showed an increase in expression upon mycorrhization of various host plants (Yuan et al. 2024). The present study shows that several genes encoding phosphatases are strongly up-regulated by Pi deficiency and that the activity of acid phosphatase released in the culture medium is also strongly increased. However, no alkaline phosphatase activity could be detected under the same condition. It is possible that the *L. bicolor* enzymes annotated as alkaline phosphatases (704119, 666564) may not have activity towards para-nitrophenyl-phosphate, the chromogenic substrate used in our assay and the assay from Yuan and colleagues (Yuan et al. 2024).

Several studies have shown that the expression of Pi transporters belonging to the PHT1 family, which are orthologues to the *S. cerevisiae* PHO84, are overexpressed in ECM fungi grown under low Pi. Examples of such regulation are found in *Tricholoma spp* (Kothe et al. 2002), *Hebeloma cylindrosporum* (Tatry et al. 2009) and *P. involutus* (Paparokidou et al. 2021). The present study shows that two *L. bicolor* genes belonging to the PHT1 family (297291 and 191924) are induced by Pi deficiency, and this was accompanied by the increase in Pi transport activity in free-living *L. bicolor* mycelium. Interestingly, a distinct *L. bicolor* PHT1 gene (600674) was strongly down-regulated by Pi deficiency, highlighting opposite regulatory profiles for different gene members of the PHT1 family.

In contrast to phosphatases, the secretion of ribonucleases by mycorrhizal fungi has, to our knowledge, not yet been reported. Secretion of ribonucleases has been reported in yeast and in yeast-like fungi belonging to the genus *Candida* and *Yarrowia*, and in the fungi *Tremella foliacea* and *T. encephala* belonging to the sub-division Agaricomycotina (Burt and Cazin 1976; Cheng and Ogrydziak 1986). Interestingly, similarly to our findings, secretion of ribonucleases in the *T. foliacea* growth medium was also regulated by Pi deficiency. Future studies should aim to examine the importance and contribution of ribonuclease activity secreted by ECM in the mobilization and transfer of Pi from soil to host roots.

Induction of acid phosphatase and Pi transporters are two of the key features of adaptation of *S. cerevisiae* to Pi deficiency, indicating that elements of the *S. cerevisiae* PHO regulon may exist in *L. bicolor*. Search for *L. bicolor* annotated genes homologous to *S. cerevisiae* genes participating in the PHO regulon unveiled several candidates for the PHO80–81–85 cyclin kinase and inhibitor complex, PHO5/PHO12/PHO11 phosphatases, as well as several genes potentially involved in the synthesis and degradation of vacuolar polyphosphate and the synthesis of inositol polyphosphate (Supplemental Table 5). Other than the PHT1 genes mentioned above, few of these *L. bicolor* genes were markedly regulated by Pi deficiency, except for a potential KCS1 homolog (638242). *S. cerevisiae* KCS1 is involved in the synthesis of the inositol pyrophosphates 5-InsP7 and 1,5-InsP8, two key molecules regulating the activity of the PHO regulon through the binding of the SPX domain. This domain is present in PHO81 and several other Pi transporters such as PHO90, PHO87 and PHO91, as well as subunits of the VTC complex engaged in vacuolar polyphosphate synthesis (Austin and Mayer 2020; Jung et al. 2018; Wild et al. 2016). Interesting is the up-regulation under low Pi of the *L. bicolor* Git1 orthologue (677598). Git1 is involved in the transport of glycerophosphoinositol and glycerophosphocholine, which can be used as sources of inositol and Pi and could also influence the levels of 5-InsP7 and 1,5-InsP8. Modulating inositol (poly)phosphates levels have been shown to impact several signaling pathways in the basidiomycete fungus *Schizophyllum commune* and similar mechanisms could also exist in *L. bicolor* (Murry et al. 2021). Collectively, these data indicate that the synthesis of inositol pyrophosphate is likely to be a central player in the response of *L. bicolor* to Pi deficiency.

In *S. cerevisae*, the activity of the master transcription factor PHO4 is not regulated transcriptionally by Pi deficiency but rather via protein phosphorylation, which controls its localization to the nucleus (Oneill et al. 1996). No clear homolog to PHO4 could be found by BLASTP searches of the *L. bicolor* genome. However, 4 genes encoding transcription factors were induced by Pi deficiency. Deciphering how these genes are transcriptionally regulated could lead to the identification of the master transcription factor involved in Pi deficiency response in *L. bicolor*.

The synthesis of the betaine lipid DGTS has been previously reported in the bacteria *Rhodobacter sphaeroides* and *Sinorhizobium meliloti*, the eukaryotic green algae *Chlamydomonas reinhardtii* and *Nannochloropsis oceanica*, as well as in the fungi *N. crassa* and *Kluyveromyces lactis* (Klug and Benning 2001; López-Lara et al. 2005; Murakami et al. 2018; Riekhof, Andre, and Benning, 2005; Riekhof et al. 2014; Riekhof, Sears, and Benning, 2005). In all these organisms, except for *C. reinhardtii*, DGTS synthesis is enhanced by Pi deficiency, where the non-phosphorus lipid replaces phospholipids such as phosphatidylcholine (PC) present in cell membranes. While previous analysis of fatty acid and lipid composition of *L. bicolor* did not detect DGTS-derived lipids in free-living mycelium (likely because Pi-rich medium was used) (Reich et al. 2009), the present study shows that both the expression of the *L. bicolor* gene orthologue to the *DGTS* synthase from *C. reinhardtii* (CrBTA1), and the amount of DGTS in *L. bicolor* lipids, are strongly enhanced under Pi deprivation. Interestingly, inspection of the transcriptomic data of *P. involutus* grown in low P media identified a BTA1 orthologue (*P. involutus* gene 16108) that was also strongly up-regulated by P deficiency (Paparokidou et al. 2021). Synthesis of DGTS is thus a strategy to economize on the internal use of P that is share with several ECM fungi. DGTS synthesis requires the conversion of phosphatidylcholine (PC) to diacylglycerol (DAG), the latter being the substrate for the DGTS synthase. Both lipase and glycerophosphoryldiester phosphodiesterase are likely to be involved in such conversion, suggesting that the up-regulation of the *L. bicolor* genes 456129 and 667823, encoding proteins with potentially such activities, could be involved in DGTS synthesis (Ngo and Nakamura 2022).

Mycocypins are inhibitors of cysteine proteases, initially characterized in the saprotrophic fungi *Clitocybe nebularis* (called clitocypin) and *Macrolepiota procera* (called macrocypin). The *L. bicolor* genome contains 19 genes encoding mycocypins, of which 7 are induced by mycorrhization (Martin et al. 2008; Plett et al. 2022). None of the *L. bicolor* mycocypins are predicted to contain a signal peptide, indicating that they are likely intracellular, although secretion via a non-canonical pathways cannot be excluded (Plett et al. 2022). Cysteine protease inhibitors synthesized from both plants and fungi can negatively affect foraging herbivores and fungivores by interfering with the digestion of food by proteases in the predator’s gut (Goulet et al. 2008; Michaud et al. 1995; Smid et al. 2013; Zhao et al. 1996). Accordingly, two specific *L. bicolor* mycocypin (311135 and 293826) showed toxic effect on the development of the nematode *Caenorhabditis sp* and acted as feeding deterrent to the springtail *Orthonychiurus* sp. (Plett et al. 2022). The induction of some mycocypins during mycorrhizal associations was suggested to be a mechanism to protect fungi from fungivores, and ultimately benefit also the colonized plant through maintenance of symbiosis (Kaneda and Kaneko 2004). Strong induction of mycocypins by Pi deficiency could thus be a mechanism triggered in *L. bicolor* to enhance defense and survival under a non-optimal growth environment and increase the success of mycorrhizal association with roots. A similar logic could also apply for the increased expression of the *L. bicolor* serine protease inhibitor (334383) belonging to the I66 family of the MEROPS protease inhibitor classification, as a similar protease inhibitor produced in the fruiting body of *Coprinopsis cinerea* was shown to be toxic to *Drosophila melanogaster* (Sabotic et al. 2012). Pi deficiency in plants is known to alter defense, increasing resistance to herbivory through activation of the jasmonate pathway (Khan et al. 2016). At the same time, Pi deficiency can weaken plant defense against some bacterial and plant pathogens (Jaskolowski and Poirier 2024; Morcillo et al. 2020). Regulation of the plant Pi deficiency response by the PHR1 master transcription factor was recently shown to be key in the promotion of root mycorrhization by AMF under Pi deficiency (Shi et al. 2021). The current work indicates that the P status of both plants and the ECM fungus *L. bicolor* is likely to dynamically contribute to alteration in plant mycorrhization outcome under Pi deficiency.

Transcriptomic analysis revealed that several genes encoding MiSSPs were induced by Pi deficiency (Table 3, Fig. 6). MiSSPs typically lack known functional protein domains and are broadly accepted as potential effector proteins participating in the establishment of the symbiotic interaction between ECM fungi and host roots (Martin et al. 2008). This was first clearly shown for MiSSP7, as *L. bicolor* transformants with reduced expression of *MiSPP7* failed to establish symbiosis with poplar roots (Plett et al. 2011). The secreted MiSSP7 peptide enter plant root cells via endocytosis, is targeted to the nucleus and interacts with the host JAZ6, a repressor of the JA signaling pathway (Plett et al. 2014). Similarly, the *L. bicolor* MiSSP8 and MiSSP7.6 were shown to be pivotal in the establishment of ECM symbiosis (Kang et al. 2020; Pellegrin et al. 2019). While expression of neither MiSSP7/7.6/8 was observed in the present transcriptome study, 5 other MiSSPs were strongly up-regulated by Pi deficiency, while one was moderately reduced in expression. Considering that Pi deficiency enhances the association of ECM fungi with host roots, it would be interesting to unravel the role of these MiSPPs, as well as of the other putative small secreted proteins found to be up-regulated under Pi deficiency (Table 3), in the establishment and maintenance of mycorrhization under low soil P conditions.

**Table 3.**
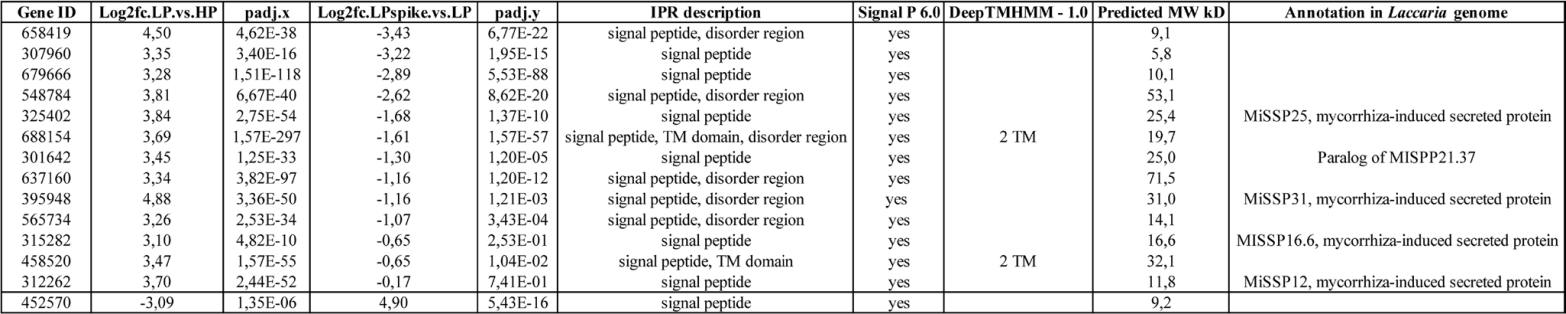
*L. bicolor* genes encoding secreted proteins up- or down-regulated under Pi deficiency

The large number of DEGs triggered by Pi deficiency reported in this work is comparable to the study of *P. involutus* by Paparokidou et al. (2021) but stands in stark contrast to the work in *L. bicolor* conducted by Ruytinx et al. (2021), the later study reporting no DEGs under low Pi treatment. The amount of total P found in mycelium grown in high and low P media between the current study (800 and 300 μmol per g dw, respectively) and the study of Ruytinx et al. (2021) (1700 and 300 μmol per g dw, respectively) are comparable. It is likely that differences in media composition and culture methods are contributing factors behind such differences in DEGs. Ruytinx et al. (2021) used a Modified Melin Norkrans medium that included malt extract and casein hydrolysate in addition to defined macro and micro-elements. In this media, the *L. bicolor* colonies produced purple pigments that were absent from the current study that used basal P5 media devoid of complex organic mixtures. Undefined compounds present in either malt extract or casein hydrolysate may have contributed to the production of purple pigments and the absence of phenotypical and transcriptomic responses to Pi deprivation, characteristics that were absent from this current study. The use of solid media containing agar in the study of Ruytinx et al. (2021) may have been another contributing factor. The type of gelling agent used to assess plant adaptations to phosphate deficiency is known to affect primary root responses, likely caused by the various concentrations of organic or inorganic contaminants (Jain et al. 2009).

## Supplementary Information

ESM 1 Supplemental Fig. 1 Ionomic analysis of free-living *L.bicolor* mycelium grown in +Pi and -Pi medium for 7 and 14 days (PDF 128 kb)

ESM 2 Supplemental Fig. 2 Amino acid sequence alignment of the *L. bicolor* protein 637045 with the *C. reinhardtii* BTA1 protein encoding the DGTS synthase. Alignment was made using the ClustalW online tool (https://www.genome.jp/tools-bin/clustalw) (PDF 342 kb)

ESM 3 Supplemental Table 1 List of genes significantly up- and down-regulated in the LP versus HP comparison with an adjusted P value ≤0.05 (XLSX 2293 kb)

ESM 4 Supplemental Table 2 List of the 12 mycocypin gene members differentially regulated upon Pi deficiency (XLSX 10 kb)

ESM 5 Supplemental Table 3 Genes without functional domains up-regulated by Pi deficiency with log2 ≥ 3 (XLSX 26 kb)

ESM 6 Supplemental Table 4 Genes without functional domains down-regulated by Pi deficiency with log_2_ ≤ −3 (XLSX 12 kb)

ESM 7 Supplemental Table 5 Expression of *L. bicolor* genes with homology to known *S. cerevisiae* genes involved in the Pi starvation response (XLSX 55 kb)

## Data Availability

The RNA-seq datasets has been deposited at the NCBI Gene Expression Omnibus under the accession number GSE302693.
