# Supplementary figures and images for: Laccaria bicolor adapts to phosphate deficiency at the developmental, transcriptional and metabolic levels
Source: Mycorrhiza. 2025 Dec 11;35(6):71. doi: 10.1007/s00572-025-01236-1 (PMC12698804; doi:10.1007/s00572-025-01236-1)

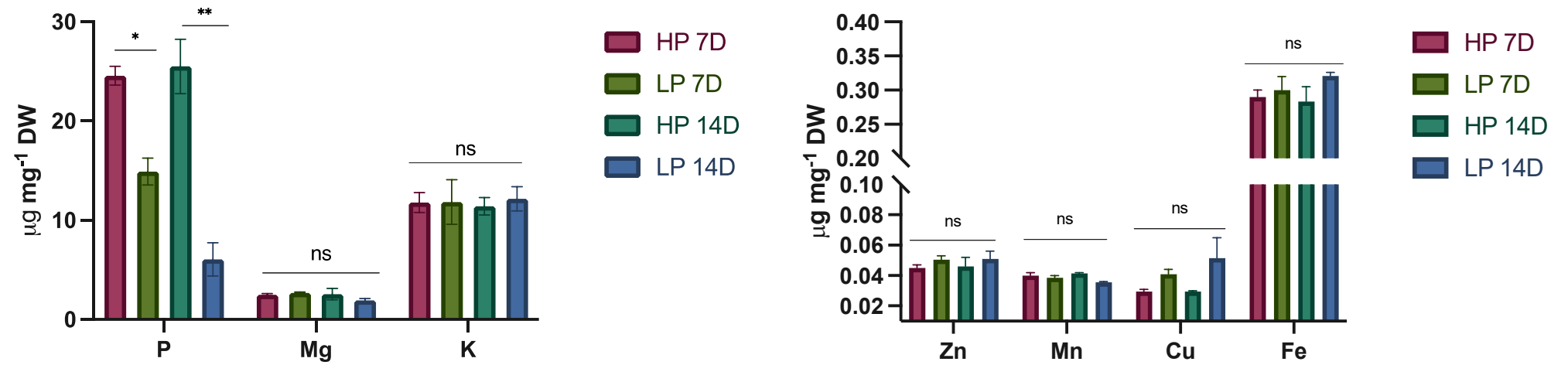

FigureS1 Ionic analysis of *L.bicolor* grown in high Pi and low Pi for 7 and 14 days.

Supplement: Supplementary file 1 — Supplemental Fig. 1 Ionomic analysis of free-living L.bicolor mycelium grown in +Pi and -Pi medium for 7 and 14 days (PDF 128 kb) [file 572_2025_1236_MOESM1_ESM.pdf]
