# Supplementary material for: Laccaria bicolor adapts to phosphate deficiency at the developmental, transcriptional and metabolic levels
Source: Mycorrhiza. 2025 Dec 11;35(6):71. doi: 10.1007/s00572-025-01236-1 (PMC12698804; doi:10.1007/s00572-025-01236-1)
Supplement: Supplementary file 2 — Supplemental Fig. 2 Amino acid sequence alignment of the L. bicolor protein 637045 with the C. reinhardtii BTA1 protein encoding the DGTS synthase. Alignment was made using the ClustalW online tool (https://www.genome.jp/tools-bin/clustalw) (PDF 342 kb) [file 572_2025_1236_MOESM2_ESM.pdf]

```
CLUSTAL 2.1 multiple sequence alignment

BTA1_Cr         --------------MGSGRDGRPASYTKKNFSLEKLKLSSMKDDLTVLRHMWFGSKKGDD
637045_Lb       MGTMVNLNSVFAPVLVIPNPTTMLAISLAALGVTFILWRMFLPQLKFIWHCFFRPLGTDN
                              :   .     : :   :.:  :    :  :*..: * :* .   *:

BTA1_Cr         HAARLESFYGPQAAAYDAFRSRFLWGRRPMLAAVAARLAERSN------LIWVDLGGGTG
637045_Lb       QKARLDKFYRGQAEVYDTTRSVLLRGRNTMLSISAAHLRQLRSSSPKKRLVWVDIGGGTG
                : ***:.**  ** .**: ** :* **..**:  **:* :  .      *:***:*****

BTA1_Cr         ENVDMMADYIDLAKFKSIYVVDLCHSLCEVAKKKAKAKGWKNVQVVEADACQFAP-----
637045_Lb       YNIELMDKYIPISSFDAIYLVDLCDSLLDVARKRFAEKGWTNVIVLCQDASEFTLPEWSQ
                 *:::* .** ::.*.:**:****.** :**:*:   ***.** *:  **.:*:

BTA1_Cr         ---PEGTATLITFSYSLTMIPPFHNVIDQACSYLS-QDGLVGVADFYVSGKYDLPLRQMP
637045_Lb       NTDPKGSVSLVTMSYSLSMIPSYYAVLDRIEHVLSPQDGLFGVVDFYTAGRLPSPHEKAI
                   *:*:.:*:*:****:***.:: *:*:    ** ****.**.***.:*:   * .:

BTA1_Cr         --------WSRRFFWRSIFDIDNIDIGPERRAYLEQKLERVWEQNTQGSIPYVPWLRAPY
637045_Lb       GGISRECGWISRWFWQIWFDFDHVHLGPARRSYLEYKFGTVKNYNGRNRFFLPFVVRIPY
                        *  *:**:  **:*::.:** **:*** *:  * : * :. :     :* **

BTA1_Cr         YVWIGR-----------------------LPSVGHALHEERVERPPMFP-----------
637045_Lb       YIWLGRPRACDVTRFCHAFEVEGGNTIGNCSPVPFKIVKEAESVPPLDIGDSIADVSSDM
                *:*:**                        ..* . : :*  . **:

BTA1_Cr         -------------------------------------PTFLYTQSWEDPEPDMEVMEINP
637045_Lb       IKAENVINITAPLSSFHYQVKNPWRLPYYEQPVHKEFRTFIYSFTWEDPFEDMKVLDLTS
                                                      **:*: :****  **:*:::..

BTA1_Cr         KDTVLTLTSGGCNALNLLVQGAG-QVVSVDCNPAQSALLELKKVAIQQLEFEDVWQLFGE
637045_Lb       EDSMLVITSAGDNALHYAIEAGPKRIHCVDMNPCQGHLLELKLAAIQSLTHDEFFSMFGK
                :*::*.:**.* ***:  ::..  :: .** **.*. ***** .***.* .::.:.:**:

BTA1_Cr         GVHPRIEELYEKKLAPFLSQTSHNFWSKRLWYFQHGLYYQGGMGKLCWVLQCLAVVLGLG
637045_Lb       GRHSNFRALLDSRISPQLSSSAYQFWRVNDDAFSSSFYMHGYSGLALRLAKIIFRLAGVT
                * *..:. * :.:::* **.::::**  .   *. .:* :*  *    : : :  : *:

BTA1_Cr         KTVKRLANAPTMEEQRRLWDSNMLIHFVKNGPKPLVWLFVKFVSLVLFNKAVLWFGGGVP
637045_Lb       KDVQALCDCDTLEDQARIWREKLRPVLLN-----------PIVVALLKSPVFCWNALGVP
                * *: *.:. *:*:* *:* .::   :::            :*  :* . .. * . ***

BTA1_Cr         GKQYALIKADGIPIENYIARTMDGVAENSHVRKQNYFYYNCLTGKFLRDNCPTYLREAAF
637045_Lb       LNQRNMILEEGT-FYDYVANTLDPLATTYLFKTGSYFYLLTLLGHYTPASCPRYLTPSGF
                 :*  :*  :*  : :*:*.*:* :* .  .:. .***   * *::   .** **  :.*

BTA1_Cr         ATLKSG------VVDNLTVSTNFFMEELKARTYTKVILMDHVDWLDMP--VANELAECLA
637045_Lb       DALKKNDGKALDAFRLHTDAIVNVLRGLSKWSLTRALVMDHIDWFSPGSVDVDEEVRELH
                 :**..      ..   * :   .:. *.  : *:.::***:**:.     .:* .. *

BTA1_Cr         KQVAPGGIVIWRSASLSPPYAELIQKAGFDVRCIRRATQG-YMDRVNMYSSFYMARRKGA
637045_Lb       RVVAPGGLVFWRSASKEPWYNKVFEKTGFKVSAVAVRQGSEAIDRVNMYRSFWRAERL--
                : *****:*:***** .* * ::::*:**.* .:     .  :****** **: *.*

BTA1_Cr         KKDN
637045_Lb       ----
```

Figure S2. Alignment of the *L. bicolor* protein 637045 with the C. *reinhardtii* BTA1 prtein encoding the DGTS synthase. Alignment was made using the ClustalW (www.genome.jp/tools-bin/clustalw).
